# Clinical Characteristics and Maternal-Perinatal Outcomes of In Vitro Fertilization Patients Referred to a Hospital in Lima, Peru

**DOI:** 10.7759/cureus.102322

**Published:** 2026-01-26

**Authors:** Christian Silva Rengifo, Renato Andree Moran Villacorta, Daniela Noel Rabines, Carlos Alberto Vergara Ascenzo, Gladys Calderón López, Máximo Vega Alcazar

**Affiliations:** 1 Department of Obstetrics and Gynecology, Hospital Nacional Guillermo Almenara Irigoyen, Lima, PER; 2 Department of Obstetrics and Gynecology, National University of San Marcos, Faculty of Medicine, Lima, PER; 3 Department of Obstetrics and Gynecology, Clínica Anglo Americana, Lima, PER; 4 Assisted Reproduction, NiuVida Specialized Center for Assisted Reproduction, Lima, PER; 5 Department of Obstetrics and Gynecology, High-Risk Obstetrics Unit, Guillermo Almenara Irigoyen National Hospital, Lima, PER

**Keywords:** in vitro fertilization, maternal morbidity, neonatal outcomes, pregnancy complications, reproductive medicine

## Abstract

Introduction: Infertility represents a major reproductive health challenge worldwide, and in vitro fertilization (IVF) has become an effective option for many couples seeking pregnancy. However, IVF has been associated with increased maternal and perinatal morbidity, with higher rates of hypertensive disorders, multiple gestations, and preterm birth. In Peru, despite the growing use of assisted reproductive technologies, national guidelines for patient selection and risk stratification remain limited.

Objective: To describe the clinical characteristics and maternal-perinatal outcomes of females with IVF-conceived pregnancies referred to the Guillermo Almenara Irigoyen National Hospital (HNGAI), a tertiary referral center in Lima, Peru.

Methods: A descriptive, observational, retrospective study was conducted between January 2024 and March 2025. A total of 42 females with IVF-conceived pregnancies managed at the High-Risk Obstetrics Unit of HNGAI were included. Maternal demographic variables, obstetric history, comorbidities, and perinatal outcomes were analyzed using Microsoft Excel 2020 (Microsoft Corporation, Redmond, WA).

Results: The mean maternal age was 41 years. Chronic hypertension, thrombophilia, and preeclampsia were among the most frequent comorbidities. Prematurity occurred in 20 neonates (46.8%). Cesarean delivery was performed in 41 patients (97.5%), and 14 pregnancies (33.3%) were multiple gestations. Fetal and neonatal anomalies were identified in six cases (14.3%), predominantly of cardiovascular origin. Thirteen neonates (31.0%) required admission to the neonatal intensive care unit (NICU). One maternal death (2.4%) occurred due to hepatic rupture secondary to severe preeclampsia.

Conclusions: IVF-conceived pregnancies managed at this national referral center were associated with a high rate of maternal and perinatal complications. These findings underscore the importance of individualized evaluation and ethical selection of IVF candidates, as well as the need for national guidelines to ensure safe reproductive care in high-risk populations.

## Introduction

According to the World Health Organization (WHO), approximately 17.5% of the global adult population is affected by infertility, with a higher prevalence observed in high-income countries [1].

Since the first successful in vitro fertilization (IVF) in 1978, assisted reproductive technologies have advanced remarkably, offering new hope to couples struggling with infertility [1]. However, several maternal and perinatal complications associated with IVF have been reported, including multiple pregnancy, preterm birth, low birth weight, hypertensive disorders of pregnancy, and placenta previa [2,3].

In September 2022, the Peruvian Ministry of Health approved a directive resolution introducing national clinical practice guidelines for the management of infertility, which include indications for IVF. Nevertheless, both these guidelines and international standards fail to clearly define/delineate contraindications or specify the medical conditions in which IVF should be avoided.

In this study, we describe the maternal and perinatal outcomes observed in our institution, a national referral hospital in Peru, to provide local data on IVF-related complications in both mothers and newborns. Our findings aim to support the development of clearer, evidence-based criteria for the safe and ethical use of IVF in high-risk populations.

## Materials and methods

This was a retrospective observational study conducted at the High-Risk Obstetrics Unit of the Guillermo Almenara Irigoyen National Hospital (HNGAI) in Lima, Peru, a tertiary referral center for complex obstetric cases. The study period extended from January 2024 to March 2025.

Clinical data were obtained from the admission registry and electronic medical records of patients admitted to the High-Risk Obstetrics Unit with pregnancies conceived through IVF. The database included demographic characteristics, obstetric history, comorbid conditions, pregnancy complications, and perinatal outcomes. Information was manually extracted by two independent investigators using a standardized data collection form to ensure completeness and accuracy. Any discrepancies were reviewed and resolved by consensus.

Women were eligible for inclusion if they (1) had a confirmed IVF-conceived singleton or multiple pregnancy, (2) were admitted and managed at the High-Risk Obstetrics Unit of HNGAI during the study period, and (3) had complete medical records available for review. Exclusion criteria included (1) spontaneous conceptions, (2) pregnancies conceived through other assisted reproductive techniques such as intrauterine insemination, and (3) incomplete or missing data preventing outcome evaluation.

The primary outcome was the assessment of maternal morbidity, defined as the presence of hypertensive disorders, gestational diabetes, or other significant complications during pregnancy. The secondary outcomes included perinatal outcomes such as preterm birth, mode of delivery, birth weight, presence of congenital anomalies, neonatal intensive care unit (NICU) admission, and perinatal mortality.

Data were entered into Microsoft Excel 2020 (Microsoft Corporation, Redmond, WA) for processing and descriptive statistical analysis. Continuous variables were summarized as means and ranges, whereas categorical variables were presented as absolute numbers and percentages. The absence of inferential statistics severely limits interpretability and prohibits causal or comparative conclusions. Data quality was verified through double entry and cross-validation to minimize transcription errors.

All patients had previously provided informed consent for the use of their anonymized clinical data for research purposes. Given the retrospective and non-interventional design, the study did not involve any additional procedures or modification of clinical care.

## Results

**Figure 1:**
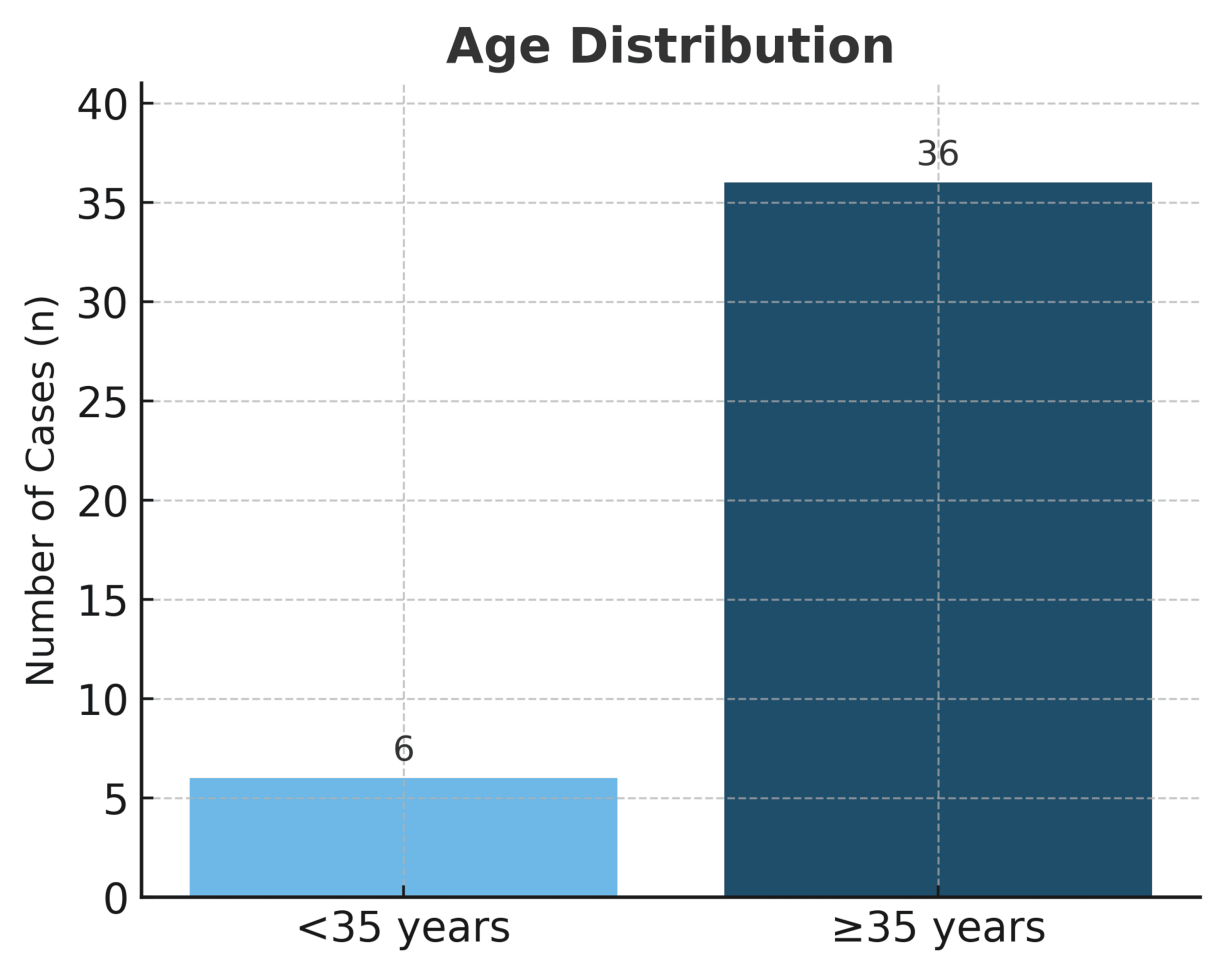
Age distribution of in vitro fertilization patients referred to Guillermo Almenara Irigoyen National Hospital, Lima, Peru.

The mean maternal age was 41 years (Figure 1), with the oldest patient being 53 years old. The mean gestational age at delivery was 35.2 weeks. Reported comorbidities included chronic hypertension (n = 3), hypothyroidism (n = 2), antiphospholipid syndrome (n = 1), stage 2 HIV infection (n = 1), multiple cervical myomatosis (n = 1), prior conventional myomectomy (n = 1), primary thrombophilia due to protein S deficiency (n = 1), and pancytopenia secondary to megaloblastic anemia (n = 1). Regarding fetal findings, one patient presented with a persistent right umbilical vein, 10 fetuses had intrauterine growth restriction, and one fetus had double outlet right ventricle with balanced atrioventricular canal and persistent left superior vena cava.

**Figure 2:**
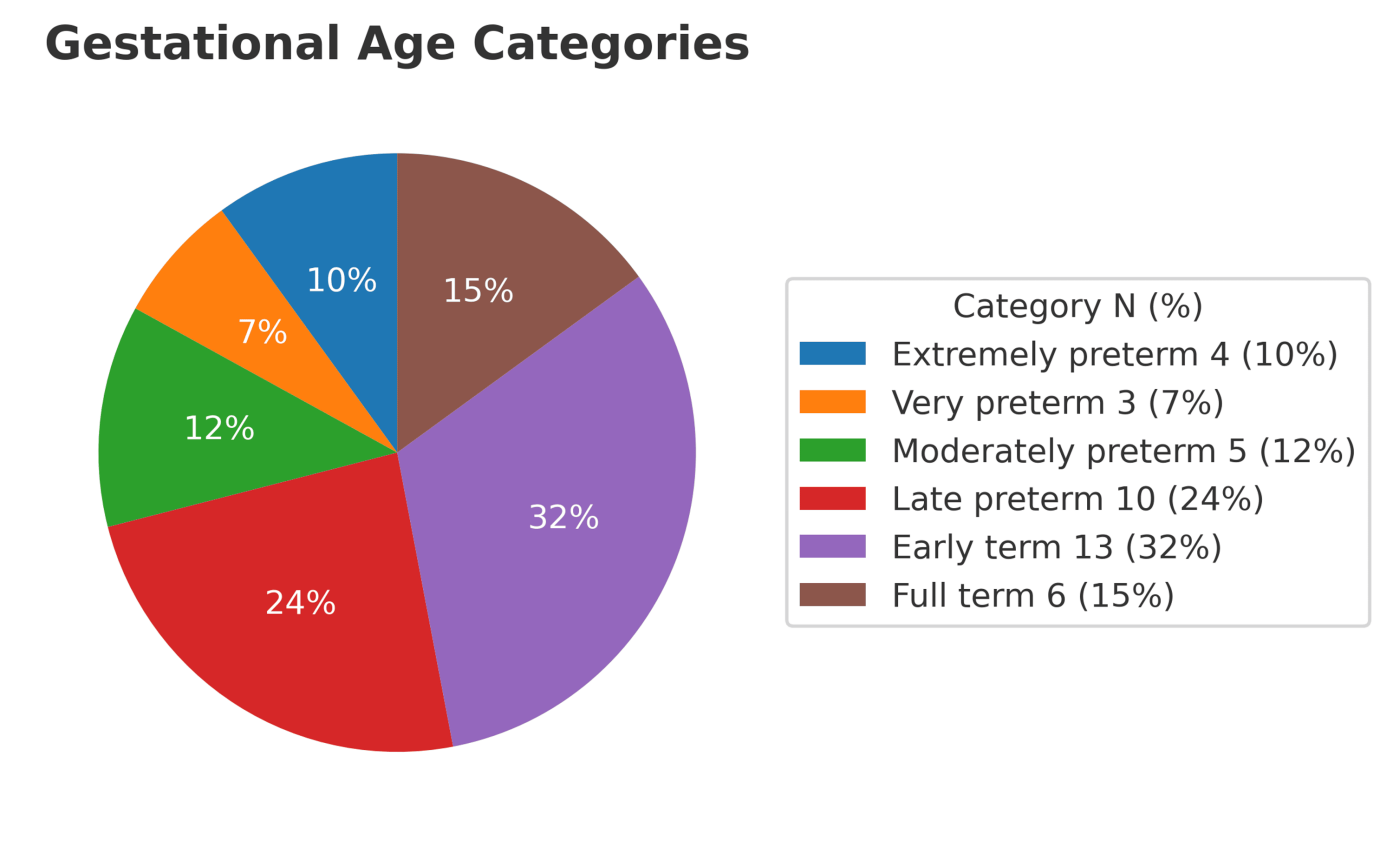
Prematurity rate among in vitro fertilization pregnancies referred to Guillermo Almenara Irigoyen National Hospital, Lima, Peru.

**Figure 3:**
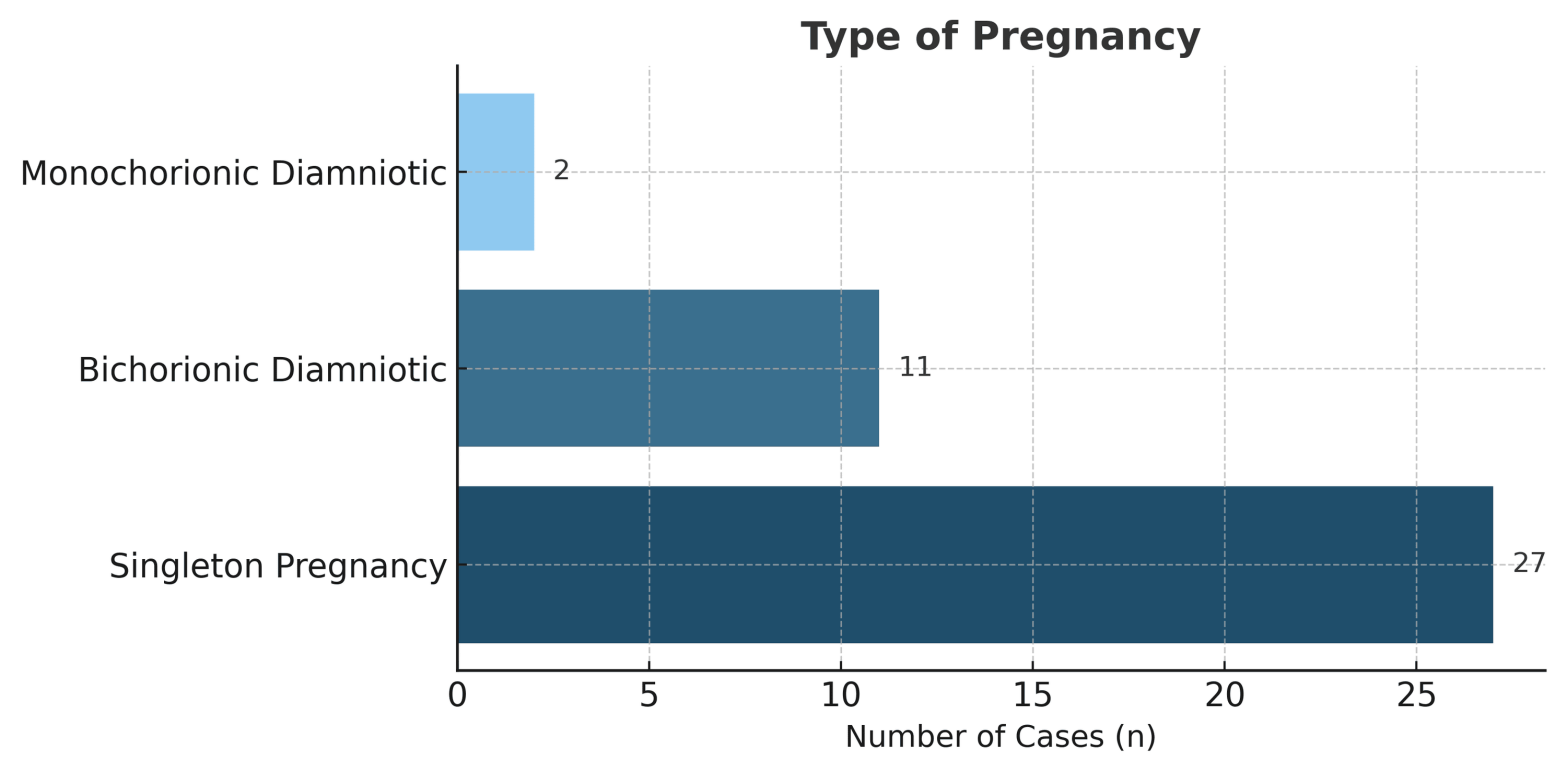
Type of gestation of in vitro fertilization patients referred to Guillermo Almenara Irigoyen National Hospital, Lima, Peru.

Among the 47 neonates, 22 were preterm (10 were late preterm, five were moderate preterm, three were very preterm, and four were extremely preterm), representing 46.81% of all births (Figure 2). The predominant mode of delivery was cesarean section in 39 cases, with only one vaginal delivery (97.5% cesarean rate). There were 14 twin pregnancies and 28 singleton pregnancies. Of the twin gestations, two were monochorionic-biamniotic, and 12 were bichorionic-biamniotic (Figure 3).

Eight patients developed preeclampsia, three of whom met criteria for severe preeclampsia. Four patients were diagnosed with gestational diabetes, and four developed chorioamnionitis at 31, 24, 32, and 26 weeks of gestation, respectively. Placenta accreta spectrum disorders were identified in two patients. Six patients required emergency cesarean delivery due to non-reassuring fetal status, five developed oligohydramnios, two experienced hemorrhagic shock (secondary to uterine atony or placenta accreta), and one patient developed intrahepatic cholestasis of pregnancy.

**Figure 4:**
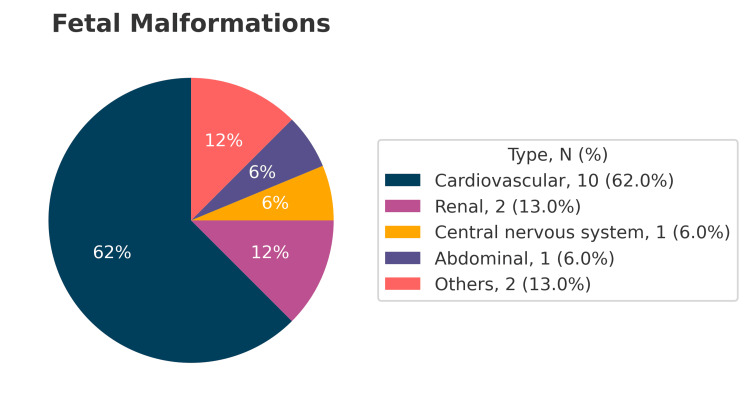
Fetal and neonatal malformations among IVF-conceived pregnancies of IVF patients referred to Guillermo Almenara Irigoyen National Hospital, Lima, Peru. IVF: in vitro fertilization.

Neonatal anomalies included persistent foramen ovale (n = 5, with one neonatal death at three days), intraventricular hemorrhage (n = 2, with one death at 31 days), umbilical hernia (n = 1), double-outlet right ventricle (n = 1, deceased at 26 days), retinopathy of prematurity (n = 3), patent ductus arteriosus (n = 3), atrial septal defect type ostium secundum (n = 2), axial hypotonia (n = 1), hydrocele (n = 1), bilateral caliectasia (n = 1), facial hemangioma (n = 1), and muscular ventricular septal defect (n = 1) (Figure 4).

Thirteen neonates required management in the NICU. One maternal death occurred due to hepatic rupture associated with severe preeclampsia. Perinatal data could not be obtained for four newborns.

## Discussion

This study presents an analysis of perinatal outcomes of patients who conceived through IVF and were referred to our hospital, including prematurity, hypertensive disorders of pregnancy, cesarean delivery rate, and multiple gestations. Consistent with existing literature, women undergoing assisted reproductive therapies tend to be of advanced maternal age. In our series, the mean age was 41 years, a factor that plays a key role in adverse outcomes. Vandekerckhove et al. reported higher rates of preeclampsia, preterm birth, cesarean delivery, and small-for-gestational-age neonates in women over 35 years old [4].

In line with our findings, several studies have demonstrated an increased incidence of perinatal complications associated with IVF. Sabban et al. [5], in a retrospective cohort of 5,773 IVF pregnancies, reported increased odds ratios for preeclampsia (OR: 1.48; 95% CI: 1.32-1.62), premature rupture of membranes (OR: 1.49; 95% CI: 1.30-1.70), chorioamnionitis (OR: 1.52; 95% CI: 1.29-1.79), and cesarean delivery (OR: 1.60; 95% CI: 1.51-1.70). Similarly, Dimanlig-Cruz et al. [6] analyzed 117,901 pregnancies, including 3,457 conceived through assisted reproductive technologies (ART), and found increased risks of stillbirth (adjusted RR: 2.26; 95% CI: 2.04-2.51), multiple pregnancy (RR: 8.95; 95% CI: 8.58-9.35), preterm birth (RR: 2.06; 95% CI: 1.98-2.14), cesarean delivery (RR: 1.44; 95% CI: 1.42-1.47), low Apgar score (RR: 1.28; 95% CI: 1.16-1.42), and neonatal intensive care unit admission (RR: 1.98; 95% CI: 1.84-2.13).

We also identified four cases of gestational diabetes mellitus (GDM). In South America, the reported incidence of GDM is approximately 11.2% (95% CI: 7.1-16.6%) [7], similar to our results. In contrast, Zhu et al. [8] reported a significantly higher incidence of GDM in IVF pregnancies (30.6%) compared with spontaneous conceptions (16%).

As documented in previous reports/publications, cardiovascular anomalies were the most frequent congenital defects in our cohort. Veeramani et al. [9], in a systematic review and meta-analysis, found that ART-conceived neonates had a higher risk of congenital anomalies compared with those conceived naturally (OR: 0.67; 95% CI: 0.59-0.76), with a 9% increase observed in cases of intracytoplasmic sperm injection (ICSI) compared with standard IVF. When classified by organ system, cardiovascular, gastrointestinal, and neurological malformations were increased regardless of technique, whereas urogenital and musculoskeletal anomalies were more frequent in ICSI conceptions.

Limitations inherent to the observational design of this study include the potential for selection bias. However, this potential bias was minimized/mitigated by the referral policy of the Almenara Health Network, whereby all pregnancies conceived via IVF must be managed at our institution. Still, this does not ensure complete coverage of all national cases. As Paulson et al. suggest, many complications may stem from the underlying conditions that led to the use of IVF rather than the procedure itself. Therapeutic channeling bias should also be considered, as IVF pregnancies are often managed with greater caution, leading to higher rates of elective cesarean deliveries, which may influence perinatal outcomes [10,11].

Future research should consider the type of endometrial preparation used for embryo transfer (fresh or frozen cycles), as emerging evidence indicates that programmed (hormone-replacement) cycles lacking a corpus luteum are associated with a higher risk of hypertensive disorders of pregnancy compared with natural or modified cycles [12,13].

In reproductive medicine, clinicians bear the ethical and professional responsibility to apply advanced technologies judiciously and based on evidence. IVF represents a major scientific achievement, fulfilling the desire for parenthood in thousands of women. However, it should not be viewed as a risk-free procedure, particularly in women with complex medical conditions or at extreme maternal ages. Current recommendations from the European Society of Human Reproduction and Embryology (ESHRE) and the American Society for Reproductive Medicine (ASRM) discourage the use of IVF in women over 50 years of age due to substantially increased maternal and perinatal risks [14]. Both societies emphasize the need for a comprehensive assessment of a patient’s physical and mental health before initiating highly complex treatments, prioritizing both safety and ethical principles [15,16].

Therefore, the role of the reproductive medicine specialist extends beyond achieving pregnancy; it encompasses ensuring that conception occurs under conditions that safeguard the health and life of both the mother and the newborn. Informed, multidisciplinary, and prudent decision-making is essential to the responsible practice of this specialty.

Overall, this study contributes to understanding the specific characteristics and risks of a population that, due to its reproductive history and clinical profile, requires specialized obstetric care. Although causality cannot be inferred, our findings provide a foundation for improving patient selection, optimizing clinical protocols, and guiding future research with more robust designs that adjust for age, comorbidities, and the type of embryo transfer.

## Conclusions

In conclusion, our results demonstrate a high rate of maternal and perinatal complications among IVF pregnancies managed at HNGAI. These findings reinforce the importance of an individualized, ethical, and safety-centered approach to reproductive care.
